# Procedural Risk Associated With Anomalous Coronary Artery Supply During Aortic Valve Replacement

**DOI:** 10.7759/cureus.92309

**Published:** 2025-09-14

**Authors:** Neha Chandna, Asmita Itani, Anjali Gaalla, Salim Surani, Munish Sharma

**Affiliations:** 1 Medicine, Victoria Heart and Vascular Center, Victoria, USA; 2 General Medicine, GP Koirala National Center for Respiratory Diseases, Tanahun, NPL; 3 Anesthesiology, Mayo Clinic, Rochester, USA; 4 Medicine, Texas A&M University, College Station, USA; 5 Pharmacy and Medicine, Texas A&M University, Kingsville, USA; 6 Internal Medicine, Pulmonary Associates, Corpus Christi, USA; 7 Clinical Medicine, University of Houston, Houston, USA; 8 Pulmonary and Critical Care, Baylor Scott & White Medical Center, Temple, USA

**Keywords:** anomalous coronary artery origin, aortic stenosis (as), right coronary sinus, surgical aortic valve replacement (savr), tavr (transcatheter aortic valve replacement)

## Abstract

Aortic stenosis (AS) is a common valvular heart disease in the elderly population. Transcatheter aortic valve replacement (TAVR) is a preferred modality for severe symptomatic AS management in elderly patients with high surgical risk. However, unusual coronary artery supply with anatomical variations may increase the procedural risk in TAVR, making surgical aortic valve replacement (SAVR) a suitable modality. This article presents a case of a 77-year-old male with severe symptomatic AS with an uncommon coronary anomaly where all three major coronary arteries originate from the right coronary sinus, posing a high risk for coronary obstruction during TAVR. The SAVR procedure was performed successfully, allowing for direct visualization and protection of the anomalous coronary ostia. This case emphasizes the crucial role of pre-procedural catheterization and surgical planning in patients with atypical coronary artery supply.

## Introduction

Aortic stenosis (AS) is the most common valvular heart disease in developed countries, and its prevalence is increasing. The recent study shows that the prevalence of AS in people more than 75 years of age is 12.4% and among them, 3.4% have severe AS [1]. If left untreated, symptomatic AS can lead to significant morbidity and mortality. Treatment decisions should consider both anatomical characteristics and comorbid factors that may affect both procedural risk and the likelihood of early and late complications [2,3]. Though the use of transcatheter aortic valve replacement (TAVR) has expanded for managing AS, surgical aortic valve replacement (SAVR) is preferred in cases with complex coronary anatomy and significant aortic valve calcification that could complicate TAVR and increase the risk of coronary obstruction [2,4]. Typically, the right coronary artery (RCA) originates from the right coronary sinus, and its course follows the right atrioventricular groove. The left coronary artery (LCA) trunk arises from the left coronary sinus. After crossing over the pulmonary trunk, the LCA gives rise to two branches: the left anterior descending (LAD) and left circumflex arteries. Anatomical variations in the origin and course of the coronary arteries are estimated to be observed in 0.3-5.6% of the population [5]. We report a rare case of a 77-year-old male with severe symptomatic AS in whom all three major coronary arteries, RCA, LAD, and circumflex artery, originated from the right side. This unusual coronary anatomy altered the management approach, leading to the selection of SAVR instead of the less invasive TAVR.

## Case presentation

A 77-year-old male patient with a past medical history of hypertension (HTN), hyperlipidemia, morbid obesity, first-degree atrioventricular block (AV block), and chronic kidney disease (CKD) presented to the hospital with exertional shortness of breath and chest pain for six months. The patient did not report any orthopnea, pedal edema, lightheadedness, or syncope. A transthoracic echocardiography (TTE) revealed severely calcified aortic valve (AV) leaflets, with an AV area of 0.56 cm², indicating critical AV stenosis. The mean gradient across the AV was 74 mmHg, and the peak velocity was 5.4 m/s. The TTE also showed moderately increased wall thickness in the left ventricle, consistent with concentric hypertrophy. There was Grade I diastolic dysfunction of the left ventricle, with elevated filling pressures. However, systolic function remained normal, with an estimated ejection fraction (EF) of 60%.

**Figure 1:**
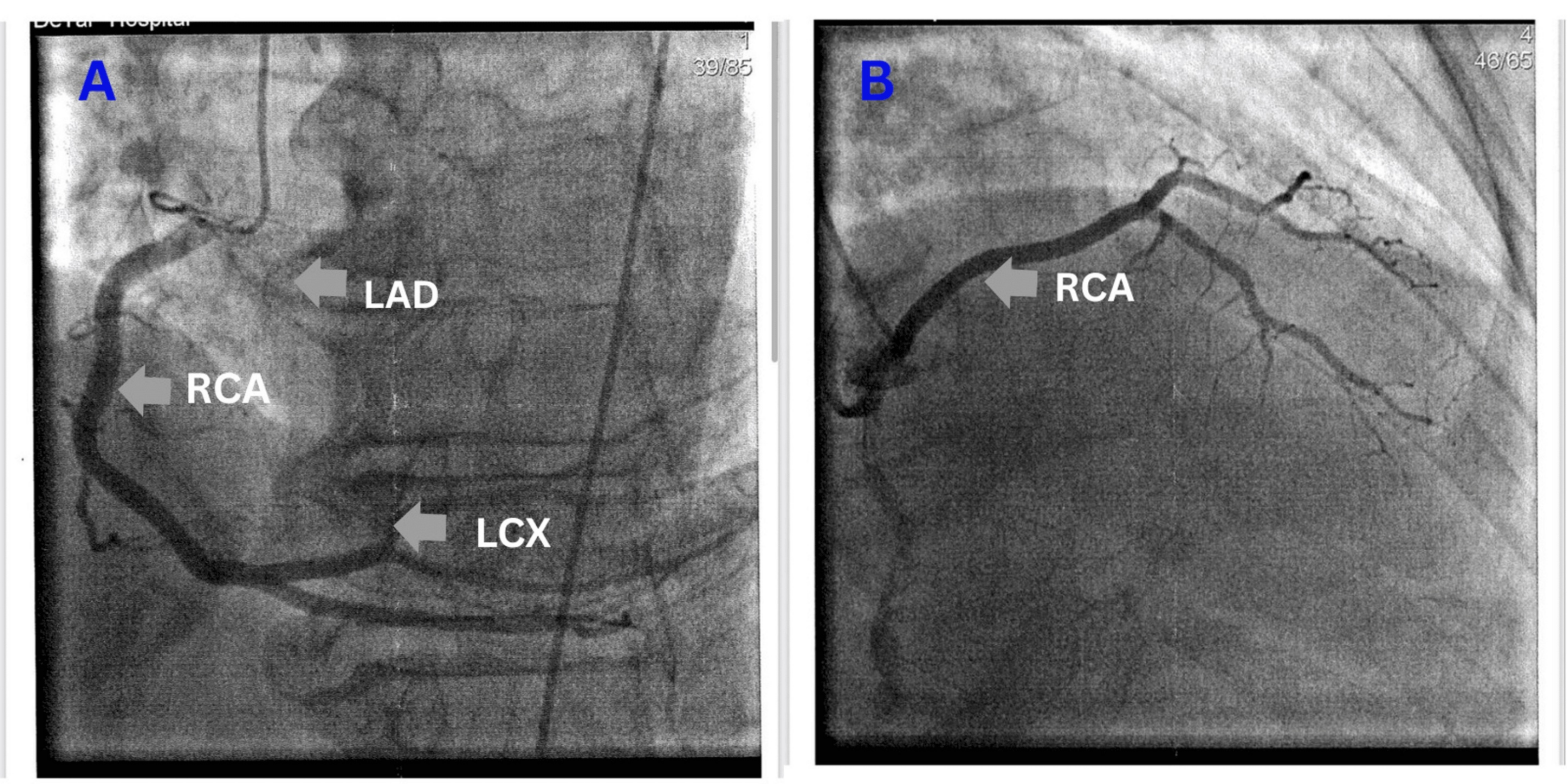
Cardiac catheterization image showing anomalous coronary arteries with a single coronary ostium originating from the right side. RCA: right coronary artery, LAD: left anterior descending artery, LCX: left circumflex artery

Cardiac catheterization was performed on January 9, 2025, revealing atypical coronary artery anatomy along with coronary artery disease (CAD). It showed a large RCA with mild intimal irregularities. The RCA gave rise to the circumflex artery. The LAD artery, which originated from the right cusp, also displayed mild intimal irregularities (Figure 1). Severe AS was confirmed, indicated by a peak-to-peak gradient of 83 mmHg, consistent with the findings from the TTE. The patient was admitted for evaluation regarding TAVR versus SAVR. Computed tomography imaging was performed on January 15, 2025, which revealed a single coronary ostium originating from the right coronary cusp, along with a high calcium score in the aorta (4330) and the coronary arteries (733). Due to a single coronary ostium originating from the right coronary cusp, TAVR was deemed high risk, leading to the decision to proceed with SAVR.

He underwent SAVR for severe non-rheumatic AV stenosis on January 24, 2025. Surgery confirmed the cardiac catheterization finding of coronary ostia all arising from the right coronary sinus. The LAD was positioned anterior to the pulmonary artery (PA), while the circumflex artery ran posterior to both the aorta and the PA. The coronary heights were adequate and well above the annulus. An endovenous vein harvest was performed as a possibility for coronary bypass if the anatomy of the coronary ostium was not favorable for placement of the AV. However, this was not required, and the coronary ostia from the right coronary sinus were all widely patent after the AV was placed and secured. Replacement was performed using a size 25 Epic Max valve. The AV and annulus were heavily calcified, necessitating debridement.

Additionally, a large, ulcerated plaque was removed from the medial wall of the mid-ascending aorta. There were no intraoperative or postoperative complications, and the patient was discharged home. Postoperative vitals included a heart rate of 62 bpm and a blood pressure of 164/72 mm Hg. However, the patient followed up a week later on January 31, 2025, with dehiscence of the lower sternal wound, which was repaired.

The patient is currently taking warfarin, amiodarone, bumetanide, ezetimibe, aspirin, hydralazine, and losartan. Warfarin and aspirin lower the risk of thromboembolic events after valve replacement surgery. Amiodarone is recommended for post-cardiac surgery patients at high risk for arrhythmias, especially those with first-degree AV block, as in our case. Bumetanide manages fluid overload in this CKD patient. Ezetimibe is prescribed for hyperlipidemia, while hydralazine and losartan optimize blood pressure in this patient with HTN. He is recovering well.

## Discussion

The study by Angelini et al. suggests the prevalence of RCA or LCA arising from the opposite Valsalva sinus was 1.07% with the use of coronary angiography. This prevalence was divided into two categories: 0.92% for the RCA originating from the left sinus of Valsalva and 0.15% for the LCA originating from the right sinus [6]. In our case, all three coronary arteries, the RCA, left circumflex artery, and LAD, arose from the right side, a very rare anomaly. The exact prevalence of this specific variation is not documented in existing literature. While most of the cases of ACAs are asymptomatic, some cases in which a coronary artery traverses between the PA and the aorta can even lead to sudden cardiac death at a young age due to extrinsic compression of the coronary artery [7]. In this case, the LAD traveled in front of the PA, and the circumflex artery traveled behind the PA and aorta, with no intervening vessels, and no compression was seen intraoperatively. However, TAVR was deemed high risk in this case due to a single coronary ostium originating from the right coronary cusp, leading the surgeon to choose SAVR.

TAVR is a minimally invasive procedure for the management of severe AV stenosis. Advancements in TAVR technology, with precise methods for annular sizing, have expanded the use of TAVR [8,9]. While the success rate of TAVR in patients with ACAs is not well established, a systematic review and meta-analysis have indicated a success rate of over 70% in a cohort of 28 patients with ACAs, all of whom experienced no immediate complications. However, the study identified acute coronary artery obstruction as the most common fatal short-term complication associated with TAVR [7,10]. Several risk factors contribute to coronary obstructions, including heavy calcification of the coronary arteries, low-lying coronary artery origins, valve misplacement, bulky leaflets, and a small sinus of Valsalva [7]. In our case, while the coronary artery origins were of adequate length, there was heavy calcification present, which significantly increased the risk of coronary artery obstruction during TAVR. Additionally, the presence of the ACA further elevated this risk. Given that SAVR is preferred for low-surgical-risk patients with severe AS who have an increased risk of coronary obstruction during TAVR, we opted for SAVR in our case [4].

Moreover, heavy calcification in the left ventricular outflow tract can lead to increased paravalvular leak, negatively impacting TAVR outcomes [11,12]. SAVR provides the opportunity to reassess coronary artery anatomy and patency while surgically removing significant calcification during the AV replacement procedure, making SAVR an ideal choice in our case [4,13].

Additionally, in our case, the removal of a large, ulcerated aortic plaque is another advantage of the open surgical approach, as it allows for the treatment of coexisting aortopathy that might remain unaddressed during a percutaneous procedure [4]. The successful implantation of an Epic Max size 25 bioprosthetic valve, along with the confirmation of patent coronary ostia afterward, demonstrates the benefits of direct visualization during SAVR, particularly in complex cases involving unusual coronary artery anatomy, as in our case.

## Conclusions

This case illustrates a rare and clinically significant coronary artery anomaly discovered during the evaluation of severe symptomatic AS. The presence of an abnormal coronary supply greatly affected the decision-making process for the AV replacement strategy. As a result, we chose to proceed with SAVR rather than the less invasive TAVR. In this patient, the combination of an anomalous coronary origin and a heavily calcified AV justified the need for open surgery. The patient’s smooth recovery reinforces the effectiveness of the surgical decision. While TAVR has become the preferred method for high-surgical-risk patients, anatomical contraindications such as this one require careful, case-by-case evaluation. This case underscores the importance of conducting a comprehensive preoperative assessment and imaging, along with individualized decisions when selecting the appropriate valve replacement method for managing severe AS in patients with unusual coronary artery supply.
